# Treatment paradigm and prognostic factor analyses of rectal squamous cell carcinoma

**DOI:** 10.3389/fonc.2023.1160159

**Published:** 2023-05-23

**Authors:** Rui Liu, Jiahui Zhang, Yinjie Zhang, Jin Yan

**Affiliations:** ^1^ Department of Clinical Medicine, Southwest Medical University, Luzhou, China; ^2^ Sichuan Cancer Hospital and Institute, Affiliated Cancer Hospital, School of Medicine, University of Electronic Science and Technology, Chengdu, China; ^3^ Respiratory Department, The First People's Hospital of Ziyang, Ziyang, China

**Keywords:** rectal cancer (RC), squamous cell carcinoma, prognosis, chemoradiotherapy (CRT), SEER (surveillance epidemiology and end results) database

## Abstract

**Background:**

Rectal squamous cell carcinoma (rSCC) is a rare pathological subtype of rectal cancer. There is no consensus on the treatment paradigm for patients with rSCC. This study aimed to provide a paradigm for clinical treatment and develop a prognostic nomogram.

**Methods:**

Patients diagnosed with rSCC between 2010 and 2019 were identified in the Surveillance, Epidemiology, and End Results (SEER) database. According to the TNM staging system, Kaplan−Meier (K-M) survival analysis was used to identify the survival benefits of different treatments in patients with rSCC. The Cox regression method was used to identify independent prognostic risk factors. Nomograms were evaluated by Harrell’s concordance index (C-index), calibration curves, decision curve analysis (DCA) and K-M curves.

**Results:**

Data for 463 patients with rSCC were extracted from the SEER database. Survival analysis showed that there was no significant difference in median cancer-specific survival (CSS) among patients with TNM stage 1 rSCC treated with radiotherapy (RT), chemoradiotherapy (CRT) or surgery (P = 0.285). In TNM stage 2 patients, there was a significant difference in median CSS among those treated with surgery (49.5 months), RT (24 months), and CRT (63 months) (P = 0.003). In TNM stage 3 patients, there was a significant difference in median CSS among those treated with CRT (58 months), CRT plus surgery (56 months) and no treatment (9.5 months) (P < 0.001). In TNM stage 4 patients, there was no significant difference in median CSS among those treated with CRT, chemotherapy (CT), CRT plus surgery and no treatment (P = 0.122). Cox regression analysis showed that age, marital status, T stage, N stage, M stage, PNI, tumor size, RT, CT, and surgery were independent risk factors for CSS. The 1-, 3-, and 5-year C-indexes were 0.877, 0.781, and 0.767, respectively. The calibration curve showed that the model had excellent calibration. The DCA curve showed that the model had excellent clinical application value.

**Conclusion:**

RT or surgery is recommended for patients with stage 1 rSCC, and CRT is recommended for patients with stage 2, and stage 3 rSCC. Age, marital status, T stage, N stage, M stage, PNI, tumor size, RT, CT, and surgery are independent risk factors for CSS in patients with rSCC. The model based on the above independent risk factors has excellent prediction efficiency.

## Introduction

Colorectal cancer (CRC) is the third most common cancer and second leading cause of cancer death in the world (1). The morbidity of rectal cancer (RC) accounts for 29% of the morbidity of CRC (2). Adenocarcinoma (AC) is the primary histological type of RC. In addition, squamous cell carcinoma (SCC) is a rare histological subtype of RC, accounting for up to 0.1-0.3% of all cases (3). Studies support differences between rectal AC (rAC) and rectal squamous cell carcinoma (rSCC) with respect to epidemiology, pathogenesis, treatment, and prognosis (4).

Compared to the more common rAC, rSCC has a worse prognosis (5, 6). rSCC and anal SCC (aSCC) have similar molecular features and are quite different from rAC (7). The pathogenesis of rSCC is currently unclear and may be related to smoking, previous exposure to radiation, chronic proctitis, squamous metaplasia, human immunodeficiency virus (HIV) infection, and human papillomavirus (HPV) infection (3).

Since the first discovery of rSCC in 1933, it has remained very rare, so some researchers have long questioned whether rSCC in fact exists (8). The following four William’s diagnostic criteria must be met for patients with rSCC (9). First, there is no continuity between the tumor and the anal squamous epithelium or the gynecological tract. Second, there is an absence of SCC at another primary site. Third, there is an absence of squamous-lined fistula in the context of inflammatory bowel disease. Fourth, there is histological confirmation.

There are presently no clinical guidelines (NCCN, ESMO, ASCO, etc.) or expert consensus on the treatment regimens for rSCC. Current regimen options have been derived based on rAC and aSCC. Most treatments are similar to those used for aSCC. In the past, surgery was the standard treatment (10). In recent years, chemoradiotherapy (CRT) has become the preferred treatment (11, 12). Most studies indicate that radiotherapy (RT) can significantly improve the overall survival (OS), local recurrence and distant metastasis of patients with rSCC (13, 14). The results of chemotherapy (CT) (5-FU plus carboplatin or mitomycin) plus radiotherapy have been shown to be satisfactory (15–20).

Nevertheless, the study of rSCC is limited by its rarity. The Surveillance, Epidemiology, and End Results (SEER) database covers approximately 28% of the United States population and provides an adequate sample for the study of rare diseases. Thus, the aim of this study was to explore the survival benefits of different treatment options for patients with rSCC through the SEER database to provide a paradigm for clinical treatment. Additionally, the independent risk factors for cancer-specific survival (CSS) in patients with rSCC were analyzed to accurately predict survival prognosis.

## Methods

### Selection of patients

**Figure 1:**
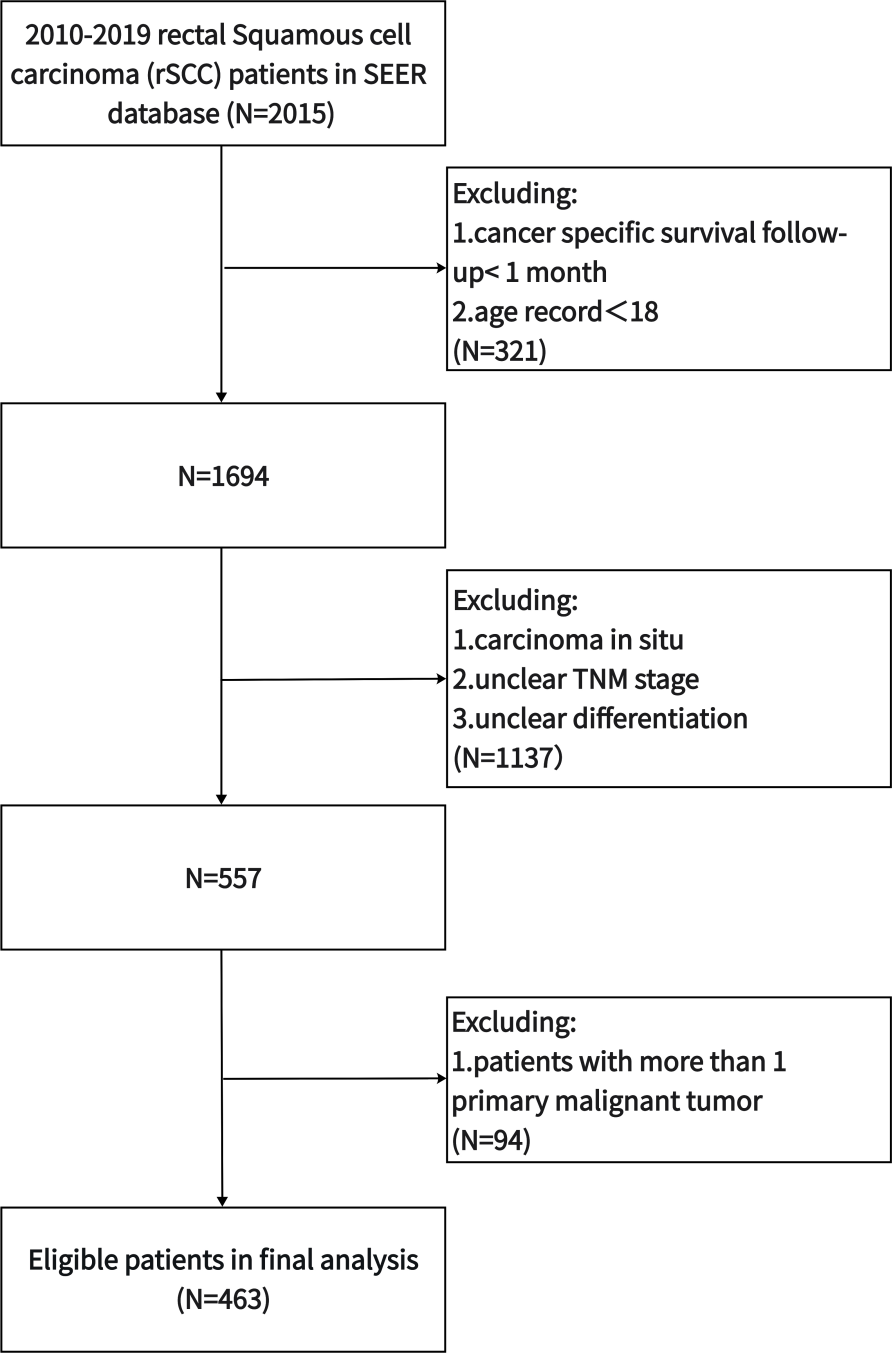
Flow chart for screening patients with rSCC from the SEER database.

Patient data were obtained with SEER∗Stat software (version: 8.4.0.1). Patients with RC diagnosed by pathology from 2010 to 2019 were included. Individual data included age, sex, race, marital status, differentiation, AJCC T stage, AJCC N stage, AJCC M stage, carcinoembryonic antigen (CEA) level, perineural invasion (PNI), tumor size, CT, RT, surgery, survival status, and survival time. The inclusion criteria were as follows (1): age at diagnosis ≥18 years old (2); patients with primary RC; and (3) rSCC identified using the International Classification of Oncology, third Revision, histological coding (8070–8077). The exclusion criteria were as follows (1): age < 18 years or survival time < 1 month (2); patients with more than one primary cancer (3); patients with missing or incomplete survival data (4); lack of TNM stage and degree of differentiation data; and (5) patients with carcinoma *in situ*. The flow chart is shown in **Figure 1**.

### Selection of variables

**Figure 2:**
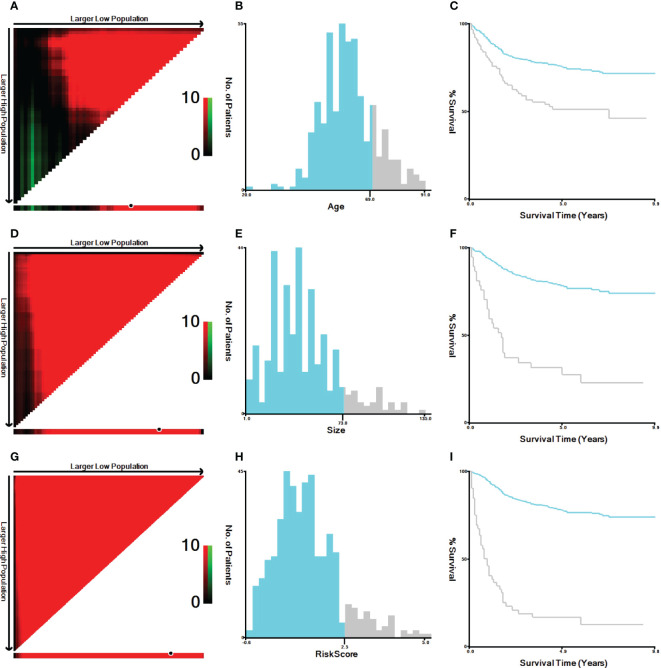
X-tile analysis of the best cutoff points for age, tumor size and risk score. **(A)** X-tile plot of age; **(B)** the cutoff point of age was highlighted using a histogram; **(C)** the distinct prognosis determined by the cutoff point was shown using a Kaplan−Meier plot of age; **(D)** X-tile plot of tumor size; **(E)** the cutoff point of tumor size was highlighted using a histogram; **(F)** the distinct prognosis determined by the cutoff point was shown using a Kaplan−Meier plot of tumor size. **(G)** X-tile plot of the risk score; **(H)** the cutoff point of the risk score was highlighted using a histogram; **(I)** the distinct prognosis determined by the cutoff point was shown using a Kaplan−Meier plot of the risk score.

The variables included in this study were age, sex, race, marital status, differentiation, TNM staging, CEA level, PNI, tumor size, CT, RT, surgery, survival status, and survival time. Unclear subvariables among marital status, CEA level, PNI and tumor size were classified as “Unknown”. The age at diagnosis was grouped as <70 years and ≥70 years. Ethnicity included White, Black, and other (American Indian/AK Native, Asian/Pacific Islander). Marital status included three subtypes: have partner, no partner and unknown. Unmarried or domestic partner and married were classified as have partner. Divorced, widowed, single (never married), and separated were classified as no partner. Tumor sizes included <73 mm, ≥73 mm, and unknown. Age and tumor size were resegmented using X-tile software, as shown in **Figure 2**. The outcome variable was CSS. CSS was defined as the time alive from diagnosis to death from cancer.

### Statistical analysis

All statistical analyses were performed with R software (version 4.2.1) on January 20th, 2023. In all statistical analyses, p < 0.05 was considered statistically significant. First, according to the TNM staging system, all patients were divided into four subtypes: TNM stage 1, TNM stage 2, TNM stage 3, and TNM stage 4. The treatment models were divided into eight subtypes: surgery, CT, RT, CRT, surgery plus RT, surgery plus CT, surgery plus CRT, and no treatment (NO). In different TNM stage subgroups, the Kaplan−Meier (K-M) method was used to analyze the efficacy of different treatment modalities. Second, to identify independent risk factors related to CSS, covariates such as age, sex, race, marital status, CEA level, T stage, N stage, M stage, differentiation, PNI, tumor size, surgery, CT, and RT were included in univariate Cox analysis. Then, the variables with statistically significant differences in univariate Cox regression analysis were included in multivariate Cox regression analysis. Third, a prognostic model for CSS was developed based on independent prognostic factors. The model was visualized as a nomogram. Fourth, receiver operating characteristic (ROC) curves of CSS at 1, 3, and 5 years were plotted, and the corresponding area under the curve (AUC) values were used to evaluate the discrimination of the model. The corresponding calibration curve was drawn to show the calibration degree of the model. Decision curve analysis (DCA) was performed to show the clinical benefit of the model. Furthermore, subgroup analysis was performed for age<70 and ≥70, have partner and no partner, N0, N1 and N2, M0 and M1, PNI negative and positive, tumor size<73 mm and ≥73 mm, RT yes and no, CT yes and no, and surgery yes and no subgroups. The K-M survival curves for each subgroup were generated.

## Results

### Clinicopathological features

According to the inclusion and exclusion criteria, 463 patients, namely, 331 female patients and 132 male patients, from the SEER database were finally included in this study. The median CSS follow-up was 59 months for females and 54 months for males (P=0.572). The female mortality rate was 26.6%, and the male mortality rate was 36.4% (P=0.037). Among females, 6 percent had well-differentiated rSCC, 44.4 percent had moderately differentiated, 47.1 percent had poorly differentiated, and 2.4 percent had undifferentiated. Among males, 9.1% had well-differentiated rSCC, 40.9% had moderately differentiated, 46.2% had poorly differentiated, and 3.8% had undifferentiated (P=0.535). Among females, 33.8% had stage T1 rSCC, 19.3% had T2, 31.7% had T3, and 15.1% had T4. Among males, 35.6% had stage T1 rSCC, 15.9% had T2, 34.8% had T3, and 13.6% had T4 (P=0.774). Among females, 66.2% had stage N0 rSCC, 29.9% had N1, and 3.9% had N2. In males, 57.6% had stage N0 rSCC, 33.3% had N1 and 9.1% had N2 (P=0.047). Among females, stage M0 rSCC was identified in 93.1% of patients, and M1 in 6.9%. Among males, stages M0 and M1 rSCC were identified in 88.6% and 11.4% of patients, respectively (P=0.118). Other information on clinicopathological features is shown in **Table 1**.

**Table 1:** Clinicopathologic features of patients with rSCC.

Characteristics	N	Female	Male	P value
		N (%)	N (%)	
Total	463	331	132	
CSS (months), median (IQR)		59 (22.5, 84)	54 (20, 84.25)	0.572
Event, n (%)				0.037
Live	327	243 (73.4%)	84 (63.6%)	
Dead	136	88 (26.6%)	48 (36.4%)	
Age, n (%)				0.775
<70	375	267 (80.7%)	108 (81.8%)	
≥70	88	64 (19.3%)	24 (18.2%)	
Race, n (%)				0.523
White	414	299 (90.3%)	115 (87.1%)	
Black	42	28 (8.5%)	14 (10.6%)	
Other	7	4 (1.2%)	3 (2.3%)	
Marital status, n (%)				0.666
Have partner	199	138 (41.7%)	61 (46.2%)	
NO partner	232	170 (51.4%)	62 (47%)	
Unknown	32	23 (6.9%)	9 (6.8%)	
Grade, n (%)				0.535
Well differentiated	32	20 (6%)	12 (9.1%)	
Moderately differentiated	201	147 (44.4%)	54 (40.9%)	
Poorly differentiated	217	156 (47.1%)	61 (46.2%)	
Undifferentiated	13	8 (2.4%)	5 (3.8%)	
T, n (%)				0.774
T1	159	112 (33.8%)	47 (35.6%)	
T2	85	64 (19.3%)	21 (15.9%)	
T3	151	105 (31.7%)	46 (34.8%)	
T4	68	50 (15.1%)	18 (13.6%)	
N, n (%)				0.047
N0	295	219 (66.2%)	76 (57.6%)	
N1	143	99 (29.9%)	44 (33.3%)	
N2	25	13 (3.9%)	12 (9.1%)	
M, n (%)				0.118
M0	425	308 (93.1%)	117 (88.6%)	
M1	38	23 (6.9%)	15 (11.4%)	
CEA, n (%)				0.493
Normal	116	79 (23.9%)	37 (28%)	
Elevated	48	37 (11.2%)	11 (8.3%)	
Unknown	299	215 (65%)	84 (63.6%)	
PNI, n (%)				0.793
Negative	192	134 (40.5%)	58 (43.9%)	
Positive	11	8 (2.4%)	3 (2.3%)	
Unknown	260	189 (57.1%)	71 (53.8%)	
Size, n (%)				0.315
<73	301	220 (66.5%)	81 (61.4%)	
≥73	39	24 (7.3%)	15 (11.4%)	
Unknow	123	87 (26.3%)	36 (27.3%)	
Radiation, n (%)				0.806
Performed	382	274 (82.8%)	108 (81.8%)	
None/Unknown	81	57 (17.2%)	24 (18.2%)	
Chemotherapy, n (%)				0.720
Performed	380	273 (82.5%)	107 (81.1%)	
No/Unknown	83	58 (17.5%)	25 (18.9%)	
Surgery, n (%)				0.188
Performed	134	90 (27.2%)	44 (33.3%)	
No	329	241 (72.8%)	88 (66.7%)	
Treatment methods, n (%)				0.927
Surgery	31	20 (6%)	11 (8.3%)	
CT	13	10 (3%)	3 (2.3%)	
RT	16	12 (3.6%)	4 (3%)	
CRT	268	196 (59.2%)	72 (54.5%)	
surgery plus CT	5	4 (1.2%)	1 (0.8%)	
surgery plus RT	4	3 (0.9%)	1 (0.8%)	
surgery plus CRT	94	63 (19%)	31 (23.5%)	
No treatment	32	23 (6.9%)	9 (6.8%)	

### Assessment of efficacy

**Figure 3:**
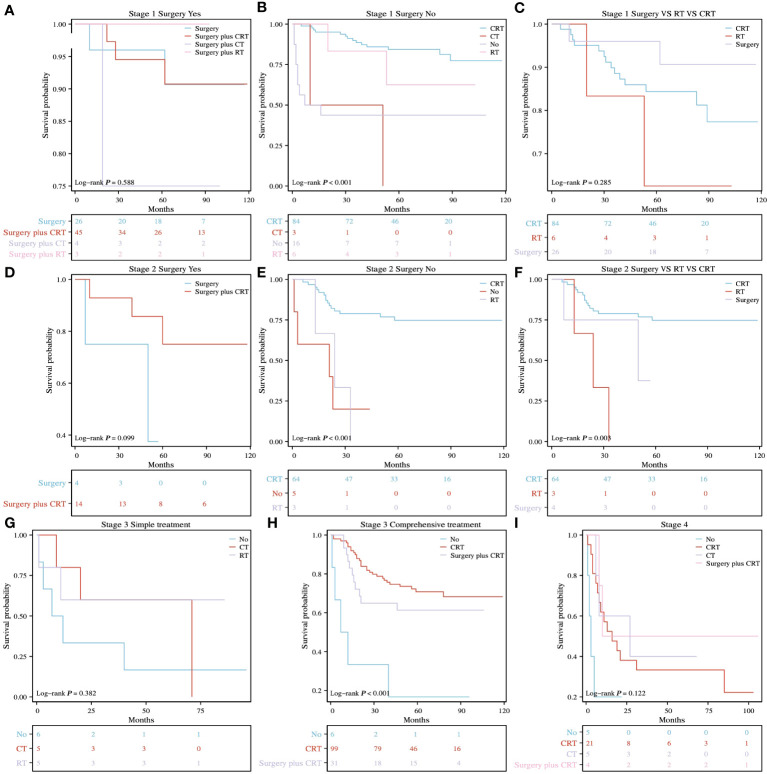
K-M survival analysis of different treatment methods for different TNM stages. **(A)** surgery vs. surgery plus CT vs. surgery plus RT vs. surgery plus CRT for stage 1, P= 0.588. **(B)** RT vs. CT vs. CRT vs. NO for stage 1, P < 0.001. **(C)** surgery vs. RT vs. CRT for stage 1, P = 0.285. **(D)** surgery vs. surgery plus CRT for stage 2, P = 0.099. **(E)** RT vs. CRT vs. NO for stage 2, P < 0.001. **(F)** surgery vs. RT vs. CRT for stage 2, P = 0.003. **(G)** RT vs. CT vs. NO for stage 3, P = 0.382. **(H)** CRT vs. surgery plus CRT vs. NO for stage 3, P < 0.001. **(I)** CT vs. CRT vs. surgery plus CRT vs. NO for stage 4, P = 0.122.

The treatment options in this study for patients with rSCC at different TNM stages are shown in **Table 2**. For patients with TNM stage 1, there was no significant difference in median CSS among those treated with surgery, surgery plus RT, surgery plus CT, and surgery plus CRT (**Figure 3A**, P= 0.588), a significant difference among those treated with CT (10 months), RT (62.5 months), CRT (64.5 months) and NO (11.5 months) (**Figure 3B**, P< 0.001), and no significant difference among those treated with surgery (68 months), RT, and CRT (**Figure 3C**, P= 0.285). For patients with TNM stage 2, there was no significant difference in median CSS among those treated with surgery and surgery plus CRT (**Figure 3D**, P = 0.099), a significant difference among those treated with RT (24 months), CRT (63 months) and NO (21 months) (**Figure 3E**, P < 0.001), and a significant difference among those treated with surgery (49.5 months), RT (24 months), and CRT (63 months) (**Figure 3F**, P = 0.003). For patients with TNM stage 3, there was no significant difference in median CSS among those treated with CT, RT, and NO (**Figure 3G**, P = 0.382) and a significant difference among those treated with CRT (58 months), surgery plus CRT (56 months) and NO (9.5 months) (**Figure 3H**, P < 0.001). For patients with TNM stage 4, there was no significant difference in median CSS among those treated with CT, CRT, surgery plus CRT and NO (**Figure 3I**, P = 0.122).

**Table 2:** Treatment methods for different TNM stages.

Characteristics(n)	Stage 1 (N=187)	Stage 2 (N=91)	Stage 3 (N=147)	Stage 4 (N=38)
Event, n (%)				
Dead	33 (17.6%)	27 (29.7%)	50 (34%)	26 (68.4%)
Alive	154 (82.4%)	64 (70.3%)	97 (66%)	12 (31.6%)
Time, median (IQR)	65 (35, 88)	58 (25.5, 90)	57 (23, 78.5)	12 (6, 61)
Paradigm, n (%)				
RT	6 (3.2%)	3 (3.3%)	5 (3.4%)	2 (5.3%)
CT	3 (1.6%)	0 (0%)	5 (3.4%)	5 (13.2%)
CRT	84 (44.9%)	64 (70.3%)	99 (67.3%)	21 (55.3%)
Surgery	26 (13.9%)	4 (4.4%)	0 (0%)	1 (2.6%)
Surgery plus CRT	45 (24.1%)	14 (15.4%)	31 (21.1%)	4 (10.5%)
Surgery plus CT	4 (2.1%)	0 (0%)	1 (0.7%)	0 (0%)
Surgery plus RT	3 (1.6%)	1 (1.1%)	0 (0%)	0 (0%)
No treatment	16 (8.6%)	5 (5.5%)	6 (4.1%)	5 (13.2%)

### Identifying independent prognostic factors

The results of multivariate Cox regression analysis showed that age, marital status, T stage, N stage, M stage, PNI, size, RT, CT, and surgery were independent prognostic factors for CSS. The results of univariate and multivariate Cox regression analyses are shown in **Table 3**.

**Table 3:** Univariate and multivariate analyses of CSS in patients with rSCC.

Characteristics	Total(N)	Univariate analysis		Multivariate analysis	
		Hazard ratio (95% CI)	P value	Hazard ratio (95% CI)	P value
Age	463				
<70	375	Reference		Reference	
≥70	88	2.268 (1.576 - 3.265)	**< 0.001**	1.611 (1.089 - 2.383)	**0.017**
Sex	463				
Female	331	Reference			
Male	132	1.402 (0.987 - 1.994)	0.059		
Race	463				
White	414	Reference			
Black	42	1.644 (1.001 - 2.700)	0.050		
Other	7	0.501 (0.070 - 3.590)	0.492		
Marital status	463				
NO partner	232	Reference		Reference	
Have partner	199	0.518 (0.358 - 0.749)	**< 0.001**	0.710 (0.480 - 1.048)	0.085
Unknown	32	0.627 (0.304 - 1.294)	0.207	0.435 (0.198 - 0.958)	**0.039**
Grade	463				
Well	32	Reference			
Moderately	201	0.893 (0.441 - 1.811)	0.754		
Poorly	217	1.093 (0.546 - 2.190)	0.802		
Undifferentiated	13	1.336 (0.448 - 3.988)	0.603		
T	463				
T1	159	Reference		Reference	
T2	85	1.029 (0.583 - 1.815)	0.922	1.121 (0.607 - 2.070)	0.715
T3	151	1.391 (0.882 - 2.194)	0.155	1.006 (0.586 - 1.726)	0.983
T4	68	3.877 (2.439 - 6.161)	**< 0.001**	1.936 (1.081 - 3.466)	**0.026**
N	463				
N0	295	Reference		Reference	
N1	143	1.983 (1.403 - 2.804)	**< 0.001**	1.653 (1.097 - 2.493)	**0.016**
N2	25	1.146 (0.527 - 2.494)	0.731	0.928 (0.401 - 2.146)	0.861
M	463				
M0	425	Reference		Reference	
M1	38	4.422 (2.877 - 6.797)	**< 0.001**	2.161 (1.316 - 3.548)	**0.002**
CEA	463				
Normal	116	Reference		Reference	
Elevated	48	1.982 (1.219 - 3.223)	**0.006**	1.463 (0.864 - 2.479)	0.157
Unknown	299	0.664 (0.448 - 0.984)	**0.041**	0.919 (0.593 - 1.423)	0.704
PNI	463				
Negative	192	Reference		Reference	
Positive	11	3.999 (1.796 - 8.906)	**< 0.001**	3.767 (1.491 - 9.517)	**0.005**
Unknown	260	1.698 (1.177 - 2.450)	**0.005**	1.213 (0.815 - 1.806)	0.342
Size	463				
<73	301	Reference		Reference	
≥73	39	4.731 (3.021 - 7.411)	**< 0.001**	2.423 (1.417 - 4.145)	**0.001**
Unknown	123	1.723 (1.171 - 2.535)	**0.006**	1.399 (0.918 - 2.132)	0.119
Radiation	463				
Yes	382	Reference		Reference	
No/Unknown	81	2.196 (1.500 - 3.216)	**< 0.001**	1.915 (1.168 - 3.143)	**0.010**
Chemotherapy	463				
Yes	380	Reference		Reference	
No/Unknown	83	2.013 (1.370 - 2.957)	**< 0.001**	2.030 (1.196 - 3.443)	**0.009**
Surgery	463				
Yes	134	Reference		Reference	
No	329	1.912 (1.239 - 2.951)	**0.003**	1.834 (1.088 - 3.091)	**0.023**

P value < 0.05 indicated by bold letters.

### Model development and validation

**Figure 4:**
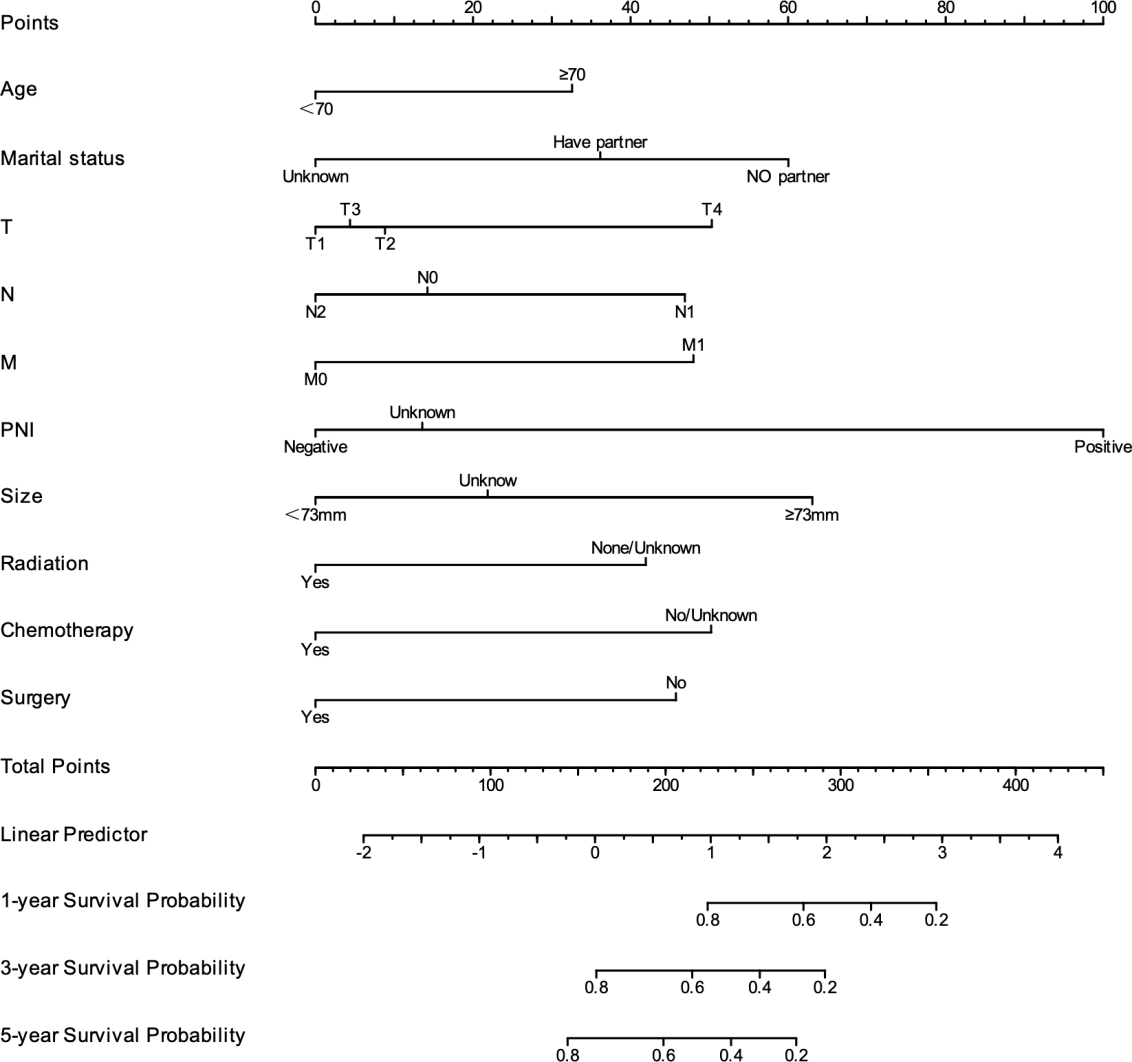
Nomogram to predict the 1-, 3-, and 5-year CSS of rSCC patients.

**Figure 5:**
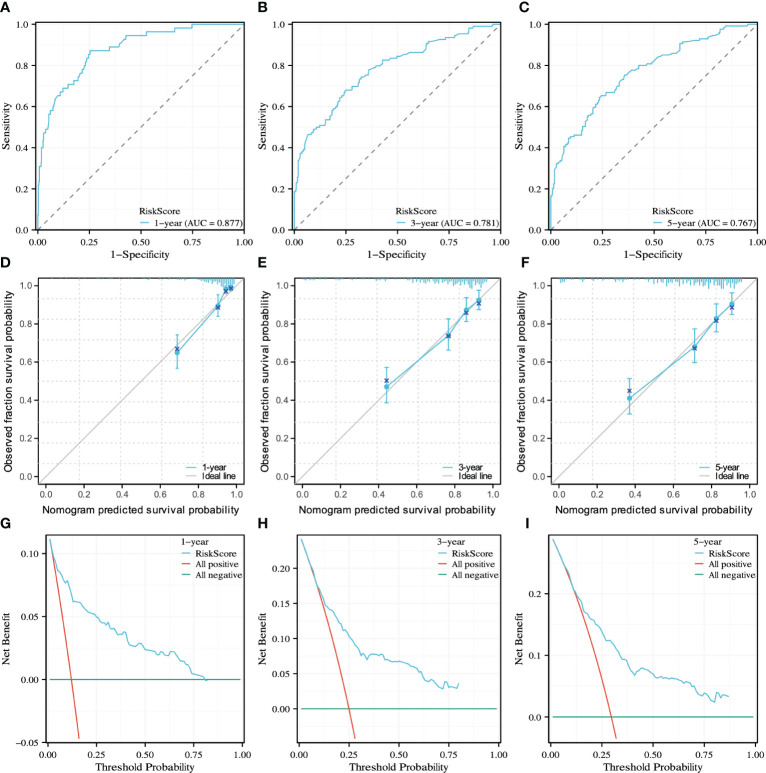
The model performance was evaluated by ROC curve, calibration curve and DCA curve. **(A-C)** ROC curves for predicting 1-, 3-, and 5-year CSS; **(D-F)** Calibration curves for predicting 1-, 3-, and 5-year CSS; **(G–I)** DCA curves for predicting 1-, 3-, and 5-year CSS.

As shown in **Figure 4**, to predict CSS in patients with rSCC, all independent prognostic factors were used to develop a prognostic model, which was visualized as a nomogram. As shown in **Figures 5A–C**, the ROC curve was drawn, and the results showed that the AUC values of CSS at 1, 3, and 5 years were 0.877, 0.781, and 0.767, respectively, indicating that the model had good discrimination. As shown in **Figures 5D–F**, the 1-, 3-, and 5-year calibration curves for CSS in patients with rSCC indicate a strong calibration of the model. As shown in **Figures 5G–I**, DCA at 1, 3, and 5 years for CSS of patients with rSCC shows that the model has high clinical benefit.

### Risk stratification

**Figure 6:**
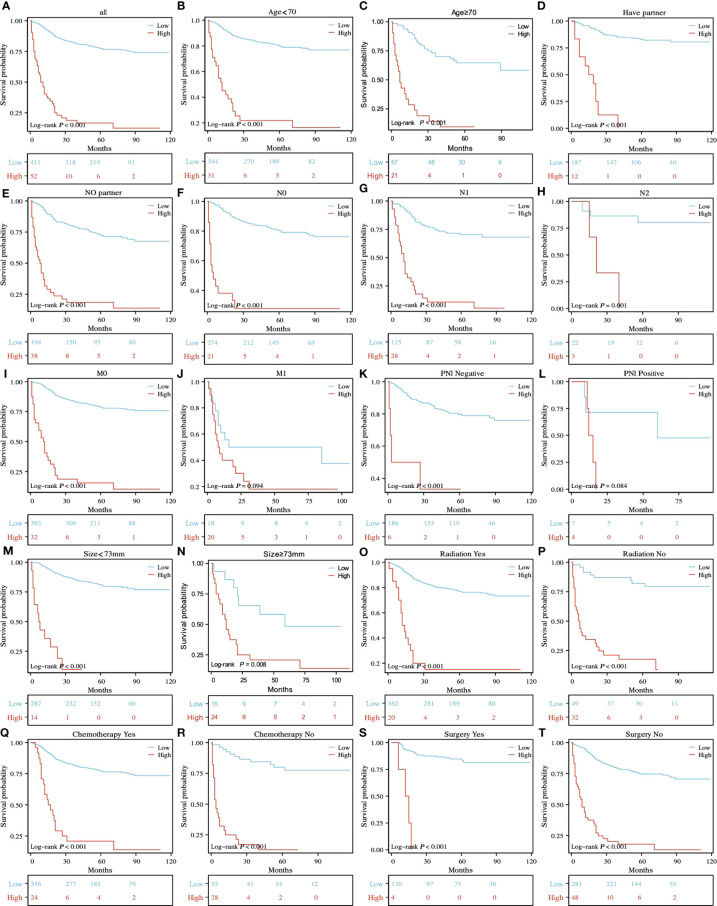
K-M survival analysis of different risk subgroups in all patients **(A)** and in the age<70 and ≥70 **(B, C)**, have partner and no partner **(D, E)**, NO, N1 and N2 **(F-H)**, M0 and M1 **(I, J)**, PNI-negative and PNI-positive z **(K, L)**, tumor size<73 mm and ≥73 mm **(M, N)**, radiation yes and no **(O, P)**, chemotherapy yes and no **(Q, R)**, and surgery yes and no **(S, T)** subgroups.

According to the prognostic model established in this study, rSCC patients can be divided into two groups: low risk and high risk. X-tile was used to determine the optimal cutoff value of the risk score, as shown in **Figures 2G–I**. Risk scores less than or equal to 2.48 were classified as the low-risk subgroup, while risk scores greater than or equal to 2.49 were classified as the high-risk subgroup. As shown in **Figures 6A–I**, **6K**, and **6M-T**, the results of Kaplan−Meier survival curves suggested that there were significant differences in survival patterns among the subgroups. In the M1 (**Figure 6J**) and PNI-positive subgroups (**Figure 6L**), the P values were 0.094 and 0.084, respectively, which may be related to the small sample size of the subgroup. With the increase in risk score, the prognosis of patients was worse. The above results suggest that the model can be used to classify rSCC patients into two groups with significantly different prognoses.

## Discussion

rSCC was first discovered in 1933 and is a rare pathological type. The occurrence of rSCC may be related to several factors. Pluripotent stem cells of mucosal endodermal origin have the ability to differentiate in multiple directions, which may lead to squamous epithelium development and malignant transformation (21). Damage to the mucosa may result in the proliferation of basal cells into squamous cells (22). HPV can induce rSCC by disrupting local cell proliferation (23). rSCC is different from AC in terms of treatment and prognosis (3, 5). rSCC tends to occur in elderly individuals and women (13).

In clinical guidelines, there is a lack of consensus on the treatment of patients with rSCC. The principles of treatment for different stages are discussed in this study. For patients with TNM stage 1, the difference between surgery and RT was not statistically significant. Adding CT or RT based on surgery had no survival benefit. Adding CT based on RT did not benefit patients. Compared with monotherapy, combination therapy was more likely to reduce tolerance and increase adverse reactions in patients. For patients with TNM stage 2, the curative effect of CRT was superior to surgery. Notably, surgery plus CRT did not increase survival benefits. For patients with TNM stage 3, CT or RT alone did not increase survival benefits compared with no treatment. The curative effect of CRT was superior to that of surgery plus CRT. For TNM stage 4 patients, CT, CRT, and Surgery plus CRT did not increase the survival benefit of patients compared to untreated patients. Schizas D and D. C. Steinemann et al. suggested that surgery is the standard treatment regimen (8, 24). In our study, surgery was significant, but only for stage 1 patients. For stage 2 and 3 patients, the survival benefit from surgery was not as good as that from CRT, which is similar to the conclusion of Kommalapati A et al. (12) Surgery in addition to CRT did not increase survival benefits, consistent with previous studies (25). The survival benefit of only surgery in stage 4 patients was not analyzed due to the limited sample size. Considering the poor quality of life of surgical patients, surgery is not recommended as a standard treatment. We recommend RT-based multimodality treatment as a paradigm for patients with rSCC, which is consistent with the findings of previous studies (19, 20, 26, 27). Surgery can be used as salvage treatment after CRT failure (18). However, salvage surgery does not seem to increase survival benefits (25). Immunotherapy has remarkable efficacy in rAC and aSCC patients with MSI-H/MSS (28–30). Neoadjuvant immunotherapy has exhibited promising results in rSCC (31). Patients may experience prolonged remission. Surgical exemption can significantly improve the quality of life of patients. In the future, multicenter phase 3 clinical trials are worth looking forward to.

In this study, univariate and multivariate Cox regression analyses were performed, and the results showed that age, marital status, T stage, N stage, M stage, PNI, tumor size, RT, CT, and surgery were independent prognostic factors for CSS. This is similar to the independent risk factor for OS (32). The 1-, 3-, and 5-year AUC values of the model were 0.877, 0.781, and 0.767, respectively, which indicated that the model had excellent discrimination. The calibration curve showed that the model had successful calibration. DCA showed that the model had outstanding clinical utility. The model constructed based on the independent prognostic factors can successfully predict the 1-, 3- and 5-year CSS of patients with rSCC. In our study, age ≥70 years was an independent risk factor, which is consistent with previous studies (12, 33). This may be because patients are more likely to not receive the full recommended treatment as they age (34). Wang et al. suggested that RC patients with partners have a better survival prognosis, which is consistent with our study (35). Distant metastasis is an important factor affecting patients’ CSS. In our study, the median CSS of stage 4 patients was only 16 months, far less than that of non-stage 4 patients. Regular cancer screening is important, and colonoscopy can be effective in the early detection of RC (36). This study found that similar to aSCC, tumor size was an independent prognostic factor in patients with rSCC. Our results agree with the study by P. Goffredo et al., which may be explained by the fact that the larger the tumor is, the more advanced the patient stage (27). However, there is no consensus on the cutoff value of tumor size.

The current study has several strengths. First, the data were obtained from the SEER database and are therefore highly reproducible. Second, more treatment options for patients with different TNM stages were explored for the first time using a large sample size, which provides valuable guidance for clinical treatment. Third, the established model has strong predictive performance and can accurately predict the survival mode of patients. Fourth, the predictors are common clinical variables, which makes the model more broadly applicable.

There are also certain limitations of this study. First, this study was based on retrospective information from the SEER database, which may have led to an inherent selection bias. Second, the clinicopathological variables included in the study were limited, such as the inability to analyze the effect of specific treatment regimens on CSS, and there was also a lack of information on immunotherapy and other treatments. Common prognostic factors such as gene expression, microsatellite status, vascular invasion, and tumor deposition were lacking, which makes the prognostic model less comprehensive. Third, this study only included information from online databases and lacked prospective data to verify the findings. It should be noted that due to the rarity of rSCC, later analyses are more likely to be based on retrospective public databases. At the same time, joint research from multiple countries and medical centers is very important for understanding rSCC.

## Conclusion

RT or surgery is recommended for patients with stage 1 rSCC, and CRT is recommended for patients with stage 2, and stage 3 rSCC. Age, marital status, T stage, N stage, M stage, PNI, tumor size, RT, CT, and surgery are independent risk factors for CSS in patients with rSCC. The model based on the above independent risk factors has excellent prediction efficiency.

## Data availability statement

Publicly available datasets were analyzed in this study. This data can be found here: https://seer.cancer.gov/.

## Author contributions

RL designed the study, conducted statistical analysis of the data, explained the results, and wrote the manuscript. JZ generated the graphs and tables. YZ proofread the manuscript for grammar. JY revised the manuscript. All authors contributed to the article and approved the submitted version.

## References

[1] Sung H Ferlay J Siegel RL Laversanne M Soerjomataram I Jemal A. Global cancer statistics 2020: GLOBOCAN estimates of incidence and mortality worldwide for 36 cancers in 185 countries. Ca-a Cancer J Clin (2021) 71(3):209–49. doi: 10.3322/caac.21660 33538338

[2] Siegel RL Miller KD Goding Sauer A Fedewa SA Butterly LF Anderson JC. Colorectal cancer statistics, 2020. CA Cancer J Clin (2020) 70(3):145–64. doi: 10.3322/caac.21601 32133645

[3] Astaras C Bornand A Koessler T. Squamous rectal carcinoma: a rare malignancy, literature review and management recommendations. ESMO Open (2021) 6(4):100180. doi: 10.1016/j.esmoop.2021.100180 34111760 PMC8193111

[4] Dyson T Draganov PV. Squamous cell cancer of the rectum. World J Gastroenterol (2009) 15(35):4380–6. doi: 10.3748/wjg.15.4380 PMC2747057 19764088

[5] Kang H O'Connell JB Leonardi MJ Maggard MA McGory ML Ko CY. Rare tumors of the colon and rectum: a national review. Int J Colorectal Dis (2007) 22(2):183–9. doi: 10.1007/s00384-006-0145-2 16845516

[6] Ozuner G Aytac E Gorgun E Bennett A. Colorectal squamous cell carcinoma: a rare tumor with poor prognosis. Int J Colorectal Dis (2015) 30(1):127–30. doi: 10.1007/s00384-014-2058-9 25392258

[7] Astaras C De Vito C Chaskar P Bornand A Khanfir K Sciarra A. The first comprehensive genomic characterization of rectal squamous cell carcinoma. J Gastroenterol (2023) 58(2):125–34. doi: 10.1007/s00535-022-01937-w PMC9876866 36357817

[8] Schizas D Katsaros I Mastoraki A Karela NR Zampetaki D Lazaridis II. Primary squamous cell carcinoma of colon and rectum: a systematic review of the literature. J Invest Surg (2022) 35(1):151–6. doi: 10.1080/08941939.2020.1824044 33021125

[9] Williams GT Blackshaw AJ Morson BC. Squamous carcinoma of the colorectum and its genesis. J Pathol (1979) 129(3):139–47. doi: 10.1002/path.1711290306 529012

[10] Ballestero Pérez A Abadía Barnó P García-Moreno Nisa F Die Trill J Galindo Álvarez J. Primary squamous cell carcinoma of the rectum: an atypical histology. Rev Esp Enferm Dig (2016) 108(12):826–35. doi: 10.17235/reed.2016.3975/2015 26911877

[11] Song EJ Jacobs CD Palta M Willett CG Wu Y Czito BG. Evaluating treatment protocols for rectal squamous cell carcinomas: the duke experience and literature. J Gastrointest Oncol (2020) 11(2):242–9. doi: 10.21037/jgo.2018.11.02 PMC7212101 32399265

[12] Kommalapati A Tella SH Yadav S Goyal G Hallemeier C Durgin L. Survival and prognostic factors in patients with rectal squamous cell carcinoma. Eur J Surg Oncol (2020) 46(6):1111–7. doi: 10.1016/j.ejso.2020.02.039 32201124

[13] Chiu MS Verma V Bennion NR Bhirud AR Li J Charlton ME. Comparison of outcomes between rectal squamous cell carcinoma and adenocarcinoma. Cancer Med (2016) 5(12):3394–402. doi: 10.1002/cam4.927 PMC5224838 27781400

[14] Guerra GR Kong CH Warrier SK Lynch AC Heriot AG Ngan SY. Primary squamous cell carcinoma of the rectum: an update and implications for treatment. World J Gastrointest Surg (2016) 8(3):252–65. doi: 10.4240/wjgs.v8.i3.252 PMC4807327 27022453

[15] Nahas CS Shia J Joseph R Schrag D Minsky BD Weiser MR. Squamous-cell carcinoma of the rectum: a rare but curable tumor. Dis Colon Rectum (2007) 50(9):1393–400. doi: 10.1007/s10350-007-0256-z 17661147

[16] Clark J Cleator S Goldin R Lowdell C Darzi A Ziprin P. Treatment of primary rectal squamous cell carcinoma by primary chemoradiotherapy: should surgery still be considered a standard of care? Eur J Cancer (2008) 44(16):2340–3. doi: 10.1016/j.ejca.2008.07.004 18707873

[17] Musio D De Felice F Manfrida S Balducci M Meldolesi E Gravina GL. Squamous cell carcinoma of the rectum: the treatment paradigm. Eur J Surg Oncol (2015) 41(8):1054–8. doi: 10.1016/j.ejso.2015.03.239 25956212

[18] Péron J Bylicki O Laude C Martel-Lafay I Carrie C Racadot S. Nonoperative management of squamous-cell carcinoma of the rectum. Dis Colon Rectum (2015) 58(1):60–4. doi: 10.1097/DCR.0000000000000218 25489695

[19] Loganadane G Servagi-Vernat S Schernberg A Schlienger M Touboul E Bosset JF. Chemoradiation in rectal squamous cell carcinoma: bi-institutional case series. Eur J Cancer (2016) 58:83–9. doi: 10.1016/j.ejca.2016.02.005 26974707

[20] Sturgeon JD Crane CH Krishnan S Minsky BD Skibber JM Rodriguez-Bigas MA. Definitive chemoradiation for squamous cell carcinoma of the rectum. Am J Clin Oncol (2017) 40(2):163–6. doi: 10.1097/COC.0000000000000126 25222072

[21] Palvio DH Sorensen FB Klove-Mogensen M. Stem cell carcinoma of the colon and rectum. report of two cases and review of the literature. Dis colon rectum (1985) 28(6):440–5. doi: 10.1007/BF02560233 2988883

[22] Michelassi F Mishlove LA Stipa F Block GE. Squamous-cell carcinoma of the colon. experience at the university of Chicago, review of the literature, report of two cases. Dis colon rectum (1988) 31(3):228–35. doi: 10.1007/BF02552552 3280272

[23] Matsuda A Takahashi K Yamaguchi T Matsumoto H Miyamoto H Kawakami M. HPV infection in an HIV-positive patient with primary squamous cell carcinoma of rectum. Int J Clin Oncol (2009) 14(6):551–4. doi: 10.1007/s10147-009-0890-7 19967495

[24] Steinemann DC Müller PC Billeter AT Bruckner T Ulrich A Müller-Stich BP. Surgery is essential in squamous cell cancer of the rectum. Langenbecks Arch Surg (2017) 402(7):1055–62. doi: 10.1007/s00423-017-1614-5 28801721

[25] Kulaylat AS Hollenbeak CS Stewart DB. Squamous cancers of the rectum demonstrate poorer survival and increased need for salvage surgery compared with squamous cancers of the anus. Dis Colon Rectum (2017) 60(9):922–7. doi: 10.1097/DCR.0000000000000881 28796730

[26] Schernberg A Servagi-Vernat S Loganadane G Touboul E Bosset JF Huguet F. Rectal squamous cell carcinoma treatment: retrospective experience in two French university hospitals, review and proposals. Cancer Radiother (2016) 20(8):824–9. doi: 10.1016/j.canrad.2016.08.128 27789176

[27] Goffredo P Robinson TJ Frakes JM Utria AF Scott AT Hassan I. Comparison of anal versus rectal staging in the prognostication of rectal squamous cell carcinoma: a population-based analysis. Dis Colon Rectum (2019) 62(3):302–8. doi: 10.1097/DCR.0000000000001205 30398999

[28] André T Lonardi S Wong KYM Lenz HJ Gelsomino F Aglietta M. Nivolumab plus low-dose ipilimumab in previously treated patients with microsatellite instability-high/mismatch repair-deficient metastatic colorectal cancer: 4-year follow-up from CheckMate 142. Ann Oncol (2022) 33(10):1052–60. doi: 10.1016/j.annonc.2022.06.008 35764271

[29] Le DT Kim TW Van Cutsem E Geva R Jäger D Hara H. Phase II open-label study of pembrolizumab in treatment-refractory, microsatellite instability-High/Mismatch repair-deficient metastatic colorectal cancer: KEYNOTE-164. J Clin Oncol (2020) 38(1):11–9. doi: 10.1200/JCO.19.02107 PMC7031958 31725351

[30] Marabelle A Cassier PA Fakih M Kao S Nielsen D Italiano A. Pembrolizumab for previously treated advanced anal squamous cell carcinoma: results from the non-randomised, multicohort, multicentre, phase 2 KEYNOTE-158 study. Lancet Gastroenterol Hepatol (2022) 7(5):446–54. doi: 10.1016/S2468-1253(21)00382-4 PMC12012850 35114169

[31] Chen G Jin Y Guan WL Zhang RX Xiao WW Cai PQ. Neoadjuvant PD-1 blockade with sintilimab in mismatch-repair deficient, locally advanced rectal cancer: an open-label, single-centre phase 2 study. Lancet Gastroenterol Hepatol (2023) 8(5):P422–431. doi: 10.1016/S2468-1253(22)00439-3 36870360

[32] Diao JD Wu CJ Cui HX Bu MW Yue D Wang X. Nomogram predicting overall survival of rectal squamous cell carcinomas patients based on the SEER database: a population-based STROBE cohort study. Med (Baltimore) (2019) 98(46):e17916. doi: 10.1097/MD.0000000000017916 PMC6867783 31725640

[33] Jarrar A Edalatpour A Sebikali-Potts A Vitello D Valente M Liska D. An up-to-date predictive model for rectal cancer survivorship reflecting tumor biology and clinical factors. Am J Surg (2020) 219(3):515–20. doi: 10.1016/j.amjsurg.2019.10.036 31703835

[34] Sarasqueta C Perales A Escobar A Baré M Redondo M Fernández de Larrea N. Impact of age on the use of adjuvant treatments in patients undergoing surgery for colorectal cancer: patients with stage III colon or stage II/III rectal cancer. BMC Cancer (2019) 19(1):735. doi: 10.1186/s12885-019-5910-z 31345187 PMC6659283

[35] Wang X Cao W Zheng C Hu W Liu C. Marital status and survival in patients with rectal cancer: an analysis of the surveillance, epidemiology and end results (SEER) database. Cancer Epidemiol (2018) 54:119–24. doi: 10.1016/j.canep.2018.04.007 29727804

[36] Areia M Mori Y Correale L Repici A Bretthauer M Sharma P. Cost-effectiveness of artificial intelligence for screening colonoscopy: a modelling study. Lancet Digit Health (2022) 4(6):e436–e44. doi: 10.1016/S2589-7500(22)00042-5 35430151

